# Response of the Class

**Published:** 1867-03

**Authors:** W. A. Grahame


### RESPONSE OF THE CLASS.

BY W. A. GRAHAME.

*Respected Professors and Honored Sirs:*—After four long months of study and application, the time has just now come for us to separate, and we must say our *adieus*; and, in behalf of my classmates, some of whom have been listening to your advice and counsel for two winters, I now appear to offer our thanks for the favors we have received at your hands. We know the ties that spring up between teachers and scholars to be strong, and unpleasant to be broken; but times for parting from tried and true friends come often "here below," and it has now come to us. We feel that you are the teachers and we the scholars, and that strong ties bind us.

"Honor is the courtesy due to kings;" but we feel that we owe that courtesy to you, and a debt of gratitude that can never be paid. But when, at parting,

> "The bitter thoughts come crowding thickly up
> For utterance, the poor, common words of courtesy
> Are such a very mockery,"

that we know not how to express our gratitude better than by simply saying, *with all our hearts we thank you.*

We are well aware that the field upon which we are just entering is both long and wide; that there is room enough yet for investigation, earnest as it may be, and that this is but just our beginning, for this is a world of mysteries that are yet unsolved—the earth, sea, air and everything in nature teem with mysterious things. Man himself is a mystery yet unknown, and he it is with whom we have to deal. He is a microcosm for study, and we fear he will never be *known* fully. Study him early and late, and he yet remains unstudied. Anatomy, physiology, chemistry, pathology, philosophy and the mechanical appliances, with their applications, constitute an array of studies that tells us that "A genius must needs go to school."

We know that time, and patience, and labor are all necessary that we attain to success in this matter. So, from this time and onward, we must be up and doing, and look to it that any great enemy to our advancement or progress be not within us.

We know, too, that this is only our first step on the "professional ladder," but we think we are starting with our "best foot foremost," and are glad that you who are above us are looking down, kindly taking us by the hands and saying, "Friends, come up higher."

But many things loom up before us, like a mirage on the plain, for which we are thankful. We are thankful that we are living in a day when Dentistry is acknowledged as a specialty in surgery; that American people are becoming educated as to what a *Dentist* ought to be, and it is now no more on a level with empiricism, but that it stands on a firm foundation, firmer than thrones of monarchs, or those who rule the world. Indeed, we think it is founded upon a rock, for rains have descended, winds have blown and beaten upon it, and it has not fallen; but it has only been made more firm, like the tree that takes deeper root as it is shaken by the winds, or more pure like the ocean that is purified by the storms that pass over it, and the tempests that stir it deeply. We are thankful that we have been told of the troubles, and trials, and vexations that await us, and how we should meet them, but still, to us, it is *unknown ground*, and will be, to a great extent, until we have trodden it with our own feet. When we walk by the sea, and endeavor to look out far upon the waters, we are filled with wonder. Now, it is not what we *see* that awes us—it is what we *don't* see. The question arises, "*What is beyond?*"

Thus it is in regard to this *unknown ground*. It will not always be those things we *see* that will bring us our difficulties, but sometimes and often it will be the things we *don't*

*see*; but we hope, by following in the paths you have pointed out to us, that we shall be better enabled to surmount these difficulties whenever they are presented. We think we shall be able to use the things we have seen and heard, to add to our structure which we are building, and not rush so rashly as the horse into battle, or so thoughtlessly as though we were looking for "the track of a serpent on a rock, or the way of a ship in midst of the sea."

You have opened the doors of Dental knowledge that we might come in, and then the windows that the pure light might shine in upon us and our every duty; and as we stand upon the threshold of the door of duty that now stands open for us to go out, it behooves us to value highly the advice and counsel of those who have grown old and wise in these things, and in going out from this place, we shall go with feelings of respect and reverence for you who have been our teachers and benefactors.

And after we have gone away from here, it will not be strange if our minds revert, time and again, to the times and scenes we have witnessed here. It is not a strange thing that a man should be patriotic and love his country, because it *helped* to make him what he is; neither is it strange that a man looks back on his "sunny hours," and school-boy days, and thinks cf his teachers, schoolmates and the "good old times" with pleasure, and these thoughts often bring the tear of regret that he cannot live his childhood o'er again. With much more pleasure shall we who have come to riper years look back upon these times and scenes. We shall be happy to feel that we are a part of that citadel of which you are the builders, and this institution the foundation stone.

Now, it is all these things for which we are thankful, and we cannot but be thankful to *you*, and to express our gratitude more fully, we must give you our best wish, and it is this: that your lives be long, your prosperity great, your influence as broad and high as heaven, and the perpetuity of this, our cherished institution, as lasting as time, and as abundant as the waves of the sea.

And, when we shall shake hands at parting; while you are bidding us a *farewell*, we hope that, at that same time and this same place, and with the same hand, you will bid us a *true* welcome into the *Brotherhood of Dentists* that now exists from sea to sea, and from the lake chain to the gulf.

*Fellow Classmates and Students:*—What shall I say to you, and how shall I say it? I have said that the time for us to separate has come, and so it has. It does not seem very long since we came here, and were formally introduced as strangers are to each other; but as we have met, day after day, in these halls, a friendship has grown up among us that I trust will not be marred nor broken by our parting. But we now go home to work, and there is a great one for us to do. We are to work for the cause of humanity; and God grant that you and I may never disgrace the work or the cause. We are going out now to fight the battle of life, to work, to act, to do; and in this we need as much courage as true soldiers, for we must "*dare to do right, dare to be true*," right between our fellowmen and ourselves, and true to those principles that are born within us, and have been so faithfully taught us. Then we ought to be sincere and true men, not *gilded*, but *gold*; not a splendid and burnished appearance outside, and a base metal within, but all the way through to the heart the same.

We are surely having advantages that those who have been before us did not possess. We can build on the foundation that other men have laid, and profit by their labors and experience. The Dental literature of *to-day* did not always exist. Dental colleges did not always exist. Many of our worthy predecessors in this work were like men who groped around in the starlight that precedes the dawn, and then—died before the sun rose. But it is "high noon" with you and me—yes, with all of us, and we ought to be aroused to the fact and *know it*. There are, everywhere, lighthouses which send a beam far out on every side, and we ought to see it, and not go groping around among the ashes and ruins of darker days, trying to find a few live, burning coals, that we may gather together and fan into a flame wherewith to light our lamps, when the world is full of light. But if all this be as clear as a sunbeam, what will it amount to unless we have *resolution* and a *purpose?* If we be men without these, we are no *men* at all; we are destitute of that manhood necessary in our calling. Therefore we say, *be resolute; have a purpose.* We read that Cæsar once having landed his army, burned his ships on the sea before their eyes to destroy the *hope* of a return. The Spartan mother said to her son going into the battle, "My son, if thy sword be too short, *add a step to it!*" They certainly had a purpose. Their motto was, "*Be true soldiers, and conquer or perish.*" We would rather see a man resolute and determined, and "stand firm as an anvil," even if he be wrong, than the one who is blown about hither and thither by every passing breeze, about whom we can have no more idea than we can in regard to the shape the clouds will assume *to-morrow.*

We think there is a new era about to be ushered in, and we think the new era for us is coming slowly, but is coming now, and is near at hand, and the duty partly falls upon you and me, now, to help it along. Let us

> "Ring out the old, ring in the new;
> Ring out the false, ring in the true."

Let us say of the death of the old era and the birth of the new one, when that time comes, as we would of the death of the old and the birth of the new year:

> "Close up his eyes, tie up his chin,
> Step from the corpse and let him in
> That standeth there alone and waiteth at the door.
> There is a new foot on the floor, my friends,
> A new face at the door, my friends,
> A new face at the door."

A few more words and I have done:

"Perhaps, henceforward, our paths may diverge widely. Each one is going his own way. If we should meet again, whether in business transactions, Dental associations, socially or otherwise, we can only hope that we be more firmly united to cherish the friendship that is the result of this winter's intercourse, and because we are engaged in the same common cause; but wherever we go, or wherever we stay, let us carry and keep with us the elements that will do *honor* to our institution and profession. And since we know we cannot live our lives over again, may we each one so live that we shall not *wish* to live again, or have to say:

> "My life has been a blighted hope,
> A blossom by the way-side thrown;
> The dreams that danced before my eyes
> In better days have flown."

But let us so live that we can render up our accounts with joy and not with grief; and in order to be able to do this, as we are now called into action, we must act like we have hearts within us, and a God o'er head.

To one and all we say—"Good Bye."

